# Editorial: Sensory processing in autism: mechanisms, impacts and interventions

**DOI:** 10.3389/fpsyt.2026.1994957

**Published:** 2026-10-05

**Authors:** Antonio Narzisi, Temple Grandin, Maddalena Fabbri-Destro, Giulio Valagussa

**Affiliations:** 1 IRCCS Stella Maris Foundation, Pisa, Italy; 2 Department of Animal Science, Colorado State University, Fort Collins, CO, United States; 3 Istituto di Neuroscienze, Consiglio Nazionale delle Ricerche, Parma, Italy; 4 University of Milano Bicocca, Monza, Italy

**Keywords:** autism, diagnosis, hypersensitivity, hyposensitivity, intervention, sensory

Sensory processing is increasingly recognized as a fundamental dimension of autism. Sensory atypicalities were already evident in Kanner’s original clinical descriptions, although they were not explicitly conceptualized as a distinct dimension of autism (1). Although hyper- and hyporeactivity to sensory input have long been described by autistic people, families, and clinicians, these features have historically received less scientific attention than social-communication differences and restricted or repetitive behaviors. Converging evidence indicates that atypical sensory perception is widespread in autism and may involve multiple sensory domains, reflecting alterations at different levels of perceptual processing (2). Yet, for many autistic individuals, sensory experiences profoundly shape everyday life. Sounds, lights, textures, smells, tastes, touch, or complex multisensory environments may be experienced as overwhelming, aversive, and sometimes even painful, affecting learning, eating, social participation, family routines, access to community settings, and quality of life.

Within this broad domain, sensory hypersensitivity deserves particular attention. Rather than being regarded as a peripheral feature accompanying the “core” characteristics of autism, hypersensitivity may interact with emotional and behavioral regulation, attention, executive functioning, motor behavior, and social engagement. Indeed, sensory alterations identified early in development have been shown to be strongly associated with subsequent emotional and behavioral difficulties, further supporting the functional relevance of sensory processing beyond the sensory domain itself (3). Understanding sensory processing may therefore provide an important window into how autistic individuals experience and navigate their environments.

First-person accounts provide a particularly compelling perspective on the intensity of sensory hypersensitivity, illustrating how sounds that may appear ordinary to others can be experienced as physically painful and profoundly threatening. As described in Thinking in Pictures: “When I was little, loud noises were a problem, often feeling like a dentist drill hitting a nerve. They also caused pain. I was scared to death of balloons popping because the sound was like an explosion in my ear” (4). Such first-person experiences underscore why sensory over-sensitivity should not be treated as a secondary or incidental aspect of autism, but as a major research priority with direct implications for well-being, participation, and quality of life.

This Research Topic emerged from this premise. In their Opinion article, Narzisi et al. highlighted the striking discrepancy between the impact of sensory sensitivities on autistic people’s lives and the attention devoted to them in research. They called for objective and developmentally informed assessment, investigation of underlying mechanisms, greater involvement of autistic people and families, and interventions and environmental adaptations capable of reducing the burden associated with sensory hypersensitivity. The contributions collected in this Research Topic approach this challenge from complementary developmental, behavioral, physiological, neurobiological, and interventional perspectives.

## Issue overview

Several contributions emphasize that there is no single or static “autistic sensory profile.” Previous work in school-aged autistic children has similarly highlighted substantial heterogeneity across sensory domains, with sensory characteristics varying in relation to cognitive functioning and differing particularly in hearing, social participation, and balance and motion (5). Extending this perspective by considering developmental change, Marani et al. examining sensory characteristics from 16 months to 14 years, identified adaptive functioning as an important correlate of sensory profiles and described a non-linear association with age. Their findings suggest a predominance of sensory seeking and auditory processing challenges during the preschool period, followed by a shift toward hypersensitivity and avoidance, particularly in visual and oral modalities, between 8 and 12 years. These results reinforce the importance of longitudinal approaches capable of capturing sensory development rather than treating sensory characteristics as stable traits.

The systematic review by Ríos Llamas et al. places hypersensitivity itself at the center of the discussion. Reviewing light and sound hypersensitivity, the authors found a literature dominated by auditory research, with evidence of reduced habituation, impaired sensory gating, and increased sensitivity to sounds, while visual hypersensitivity remains comparatively understudied. Their review also identifies a major representational gap: children and males predominate, whereas autistic females and adults remain substantially underrepresented. Future sensory research must therefore embrace a lifespan perspective and address gender imbalance while increasing ecological validity.

An equally important challenge concerns how sensory processing should be measured. Chounan and Suzuki found clear differences between autistic and typically developing adolescents in caregiver-reported sensory characteristics but no significant group difference in active-touch texture/material matching. Objective tactile performance was also not consistently associated with caregiver-reported sensory reactivity. This apparent discrepancy is informative: everyday hypersensitivity may not be reducible to elementary perceptual discrimination, and active exploration may allow individuals to regulate sensory input in ways that are not possible when sensory events are unpredictable or uncontrollable.

Similarly, Brouche et al. investigated electrodermal responses to graded unisensory and multisensory stimulation and identified an atypical pattern characterized by reduced tonic and phasic electrodermal activity in autistic children and adolescents. The coexistence of physiological hypoactivity with clinically reported sensory difficulties illustrates the complexity of the relationship between subjective experience, autonomic reactivity, perception, and behavior. Multimethod approaches combining subjective reports, behavioral measures, and physiological indices are therefore essential.

Other contributions explore possible mechanisms and correlates. Varbanov et al. using psychophysical assessment in a non-clinical adult sample found that both autistic and ADHD traits were associated with reduced orientation sensitivity, while anxiety more specifically mediated ADHD-related associations, suggesting that superficially similar sensory features across neurodevelopmental dimensions may reflect partly distinct mechanisms. Tezcan et al. using MRI identified differences in optic chiasm morphology and other neuroanatomical parameters in autistic children and explored their relationships with clinical characteristics. Although such findings require replication, they illustrate the potential value of connecting sensory phenotypes with neurobiological measures.

Importantly, sensory processing does not operate in isolation. Ji et al. found that components of executive functioning, particularly emotional and behavioral regulation, mediated associations between sensory processing patterns and social responsiveness. These findings invite a more integrated interpretation of autistic behavior: difficulties observed in social situations may sometimes be influenced by the regulatory demands imposed by sensory environments. Consistent with the broader functional impact of sensory processing, Zhang et al. showed that sensory sensitivity and avoidance, together with social-behavioral factors, uniquely predicted parental caregiving burden. Sensory difficulties therefore extend beyond the individual, affecting family functioning and caregiver well-being.

The relationship between sensory processing and everyday functioning is particularly evident in feeding. Riccio et al. found that food selectivity was significantly associated with tactile, gustatory, and olfactory sensitivities, whereas behavioral profile, intellectual functioning, and autism symptom severity were not significantly associated with food selectivity. These findings support the view that selective eating in autism may, for some individuals, be fundamentally rooted in sensory experience rather than representing merely a behavioral problem.

The Proyecto Wiñay protocol by Yago et al. further broadens this perspective by proposing an integrated investigation of sensorimotor processing, dietary patterns, nutritional status, and gut microbiota in Ecuadorian autistic children and adolescents. Its multidimensional and culturally contextualized approach highlights the importance of examining sensory phenomena alongside biological, nutritional, socioeconomic, and environmental factors.

A further cluster of contributions addresses motor and sensorimotor functioning. Cao et al. found that lower family income was associated with a greater risk of probable developmental coordination disorder among autistic children, reminding us that functional outcomes emerge from interactions between neurodevelopmental characteristics and environmental resources. In their systematic review and meta-analysis, Feng et al. found that exercise interventions, particularly structured land-based sports, improved balance, locomotor, and object-control skills. In a second systematic review and meta-analysis, Feng et al. reported that physical play-based games significantly improved social behavior, while evidence regarding executive functioning remained less conclusive. Complementing these findings, Wu et al. reported that basketball training might enhance executive functioning in autistic children. Collectively, these studies support an embodied view in which movement, sensorimotor experience, cognition, and social participation are interconnected.

The Research Topic also addresses the crucial question of intervention. The systematic review and meta-analysis by Lyu et al. including 16 studies and 1,319 participants, reported improvements in sensory integration measures and autism-related behaviors following sensory integration-based interventions, while also identifying intervention duration as a contributor to heterogeneity. These findings are encouraging but underscore the need for rigorous trials and precise characterization of intervention content and dosage.

Al-Ghabban et al. in a randomized trial of low-frequency repetitive transcranial magnetic stimulation, reported improvements across cognitive, social, and sensory measures, including reduced sensory hypersensitivity. These preliminary results point toward possible neuromodulatory approaches, although larger studies, replication, and longer-term assessment are necessary before clinical implications can be established.

Importantly, intervention need not always mean changing the autistic individual. The SENSE project by Maggio et al. examined the integration of a multisensory environment into a public school as a structured sensory break. Despite the small case series, the intervention was feasible and safe and was associated with improved sensory regulation and reduced challenging behaviors. This contribution highlights an essential principle: environments themselves can be modified to reduce sensory burden.

This principle resonates strongly with the Perspective by Chilà et al. who reconsider sensory-based visual attention through a neurodiversity-informed framework, shifting the emphasis from normalization toward adaptive support. Behaviors traditionally interpreted primarily as deficits may, in some cases, represent adaptive responses to sensory demands. Supporting autistic people should therefore not automatically mean increasing tolerance of intrinsically aversive sensory experiences; it may instead require creating environments that respect individual sensory needs.

## Conclusion

Taken together, the contributions to this Research Topic support a central message: sensory processing—and hypersensitivity in particular—should move from the periphery toward the center of autism research and clinical practice. Sensory experiences can influence eating, attention, emotional and behavioral regulation, motor functioning, social participation, family life, and access to educational and community environments. At the same time, this collection demonstrates that sensory hypersensitivity is not a unitary phenomenon: subjective experience, caregiver observation, psychophysical performance, physiological responses, neurobiological measures, developmental stage, and environmental context do not necessarily map onto one another in a simple way.

Consistent with this perspective, sensory over-sensitivity in autism should be regarded as a top priority for future research, with particular attention to experiences that are painful, unpredictable, or difficult to control in everyday environments.

Future research should therefore adopt longitudinal, multimethod, and ecologically valid approaches; include autistic females and adults more systematically; integrate objective measures with autistic people’s lived experiences; and evaluate sensory outcomes as meaningful endpoints in intervention studies. Equally importantly, research should distinguish between helping autistic individuals regulate unavoidable sensory input and modifying environments that impose unnecessary sensory burden.

The field may therefore need to move beyond asking simply how sensory processing is altered in autism toward asking how sensory experiences shape autistic people’s everyday lives and how sensory distress can be reduced while respecting individual sensory needs. In this sense, the contributions gathered in this Research Topic represent both a response to the call for greater scientific attention to sensory processing in autism and a starting point for a broader research agenda, one in which sensory hypersensitivity is no longer regarded as a peripheral feature of autism, but as a fundamental dimension of the autistic experience.
